# Emergent synchronous beating behavior in spontaneous beating cardiomyocyte clusters

**DOI:** 10.1038/s41598-021-91466-y

**Published:** 2021-06-04

**Authors:** Kazufumi Sakamoto, Yoshitsune Hondo, Naoki Takahashi, Yuhei Tanaka, Rikuto Sekine, Kenji Shimoda, Haruki Watanabe, Kenji Yasuda

**Affiliations:** 1 Department of Pure and Applied Physics, Graduate School of Advanced Science and Engineering, Waseda University, Tokyo, 169-8555 Japan; 2 Department of Physics, School of Advanced Science and Engineering, Waseda University, Tokyo, 169-8555 Japan

**Keywords:** Biophysics, Dynamic networks

## Abstract

We investigated the dominant rule determining synchronization of beating intervals of cardiomyocytes after the clustering of mouse primary and human embryonic-stem-cell (hES)-derived cardiomyocytes. Cardiomyocyte clusters were formed in concave agarose cultivation chambers and their beating intervals were compared with those of dispersed isolated single cells. Distribution analysis revealed that the clusters’ synchronized interbeat intervals (IBIs) were longer than the majority of those of isolated single cells, which is against the conventional faster firing regulation or “overdrive suppression.” IBI distribution of the isolated individual cardiomyocytes acquired from the beating clusters also confirmed that the clusters’ IBI was longer than those of the majority of constituent cardiomyocytes. In the complementary experiment in which cell clusters were connected together and then separated again, two cardiomyocyte clusters having different IBIs were attached and synchronized to the longer IBIs than those of the two clusters’ original IBIs, and recovered to shorter IBIs after their separation. This is not only against overdrive suppression but also mathematical synchronization models, such as the Kuramoto model, in which synchronized beating becomes intermediate between the two clusters’ IBIs. These results suggest that emergent slower synchronous beating occurred in homogeneous cardiomyocyte clusters as a community effect of spontaneously beating cells.

## Introduction

Synchronization of beating interval is one of the essential dynamic characteristics of cardiomyocytes to provide the blood pumping function of the heart. Goshima explained this coordinated synchronous behavior as the faster firing regulation of heart beating^1^. This conducting regulatory mechanism can also suppress the spontaneous beating of cells such as Purkinje fibers and atrioventricular node (AVN) as subsidiaries to follow the conducting spontaneous contraction impulses from the upstream sinoatrial node (SA node) with its faster beating as the dominant pacemaker^2,3^. Hence, although Purkinje fibers have the ability to spontaneously fire at a lower beating rate when they are isolated, the contraction impulse from the upstream SA node having faster beating always stimulates Purkinje fibers and suppresses their ability to beat at their own speed. Finally, Purkinje fibers follow the faster beating rate of the upstream SA node. This faster firing regulation of heart beating is now called “overdrive suppression”^4^.

Overdrive suppression is explained electrophysiologically by the following simple and straightforward mechanism with several regulatory factors^5–8^: An increase of intracellular sodium ions of a cardiomyocyte, which is caused by the depolarizing sodium ion currents of adjacent cardiomyocytes with faster spontaneous beating, stimulates the activity of the Na-K pump to expel more sodium from the cell in exchange for more potassium entering. This increased hyperpolarizing potassium current offsets the required depolarizing current, and hence membrane potential becomes more negative to prevent depolarization for the spontaneous beating. However, this interpretation alone cannot explain the mechanisms of human SA node dysfunction^9^ or phenomena in heart tissues such as subclinical atrial fibrillation^10^ and fatigue of the His–Purkinje system^11^. In this context, the following question arises: can overdrive suppression dominate and ensure that the synchronized beating interval of homogeneous cardiomyocyte clusters matches that of the fastest-beating constituent cell?

Mathematical models of synchronization phenomena such as the Kuramoto model^12^ or its extensions, describing synchronization phenomena caused by the interaction of oscillators, have played an essential role in explaining biological phenomena such as cardiomyocyte synchronization^13^, neural networks^14^, and circadian rhythms^15^. In these models, each oscillator is described only by its phase, and all oscillators are equally coupled. These simplifying assumptions have helped to understand the synchronization of many-body nonlinear dynamic systems.

In our previous on-chip constructive cell network studies, the synchronization behavior of spontaneously beating single cardiomyocytes during stepwise cell-to-cell connection to form cell networks could not be explained only by a one-way upstream-to-downstream regulatory mechanism. In particular, the behavior of equal two-way network communication of cardiomyocytes in their small networks did not show the overdrive suppression phenomenon, but followed a transition from unstable cardiomyocytes to more stable ones, even though the beating of the more stable cardiomyocytes were slower than those of the unstable ones^16,17^. In addition, the tendency of entrainment of cardiomyocyte synchronization and synchronized beating intervals was shown to depend on the number of cardiomyocytes in the network^18,19^. These experimental results showing a regulatory mechanism that beating cardiomyocyts having larger IBI fluctuations tend to synchronize to the cardiomyocytes having smaller IBI fluctuations (the lower fluctuation regulation of synchronized heart beating) have been examined as in silico model simulations, which successfully explained the experimental results by employing the “fluctuation–dissipation theorem” in the phase-couple synchronization model^20^.

For cell-to-cell synchronization studies, a sufficient number of cells with a homogeneous phenotype are required to simplify interpretation of the experimental results. However, one of the problems of primary cell-based research is quality control of the cells. Recent technological progress in the field of regenerative medicine has enabled us to provide cardiomyocytes of a single phenotype derived from pluripotent stem cells having the ability to beat just the same as in vivo cardiomyocytes and to substitute for cardiomyocytes that are absent for some reason such as aging^21–23^. Features including the action potential of cardiomyocytes derived from stem cells have also been widely studied to support this regenerative medicine approach^24,25^.

Cardiomyocyte clustering technologies have been developed by many groups to induce the efficient differentiation of induced pluripotent stem cells (iPS cells)^26^, or for drug evaluation in an environment more closely resembling in vivo conditions^27,28^. For example, the production of cardiomyocytes from a small cluster of embryoid bodies using a fabricated micro-aggregation device was reported^29^.

In this study, we examined the origin of the synchronization behavior of cardiomyocytes in clustered form in order to understand the dominant rule determining beating synchronization, in terms of how the synchronized beating intervals are generated. We examined this from the perspective of overdrive suppression, by comparing the beating characteristics of clusters and isolated single cells experimentally via a constructive approach. The results indicated that the synchronized beating intervals of clusters did not match the interval of the constituent cardiomyocyte with the fastest beating, and hence overdrive suppression cannot explain the synchronization of clusters. We also compared the synchronization behavior of two cardiomyocyte clusters after their physical attachment and the further changes after their separation again. The results showed that the synchronized beating was slower than that of either of the two clusters, which could also not be explained by the faster firing regulation or well-known mathematical phase synchronization models like the Kuramoto model^12^. These results indicate the existence of a hidden rule of competitive synchronization behavior of cardiomyocyte clusters, not only by a one-way upstream-to-downstream regulatory mechanism but also in a manner dependent on the cell-network size and a geometry-dependent selection mechanism as an emergent community effect.

## Results and discussion

### Cluster formation in concave agarose microstructure well

**Figure 1:**
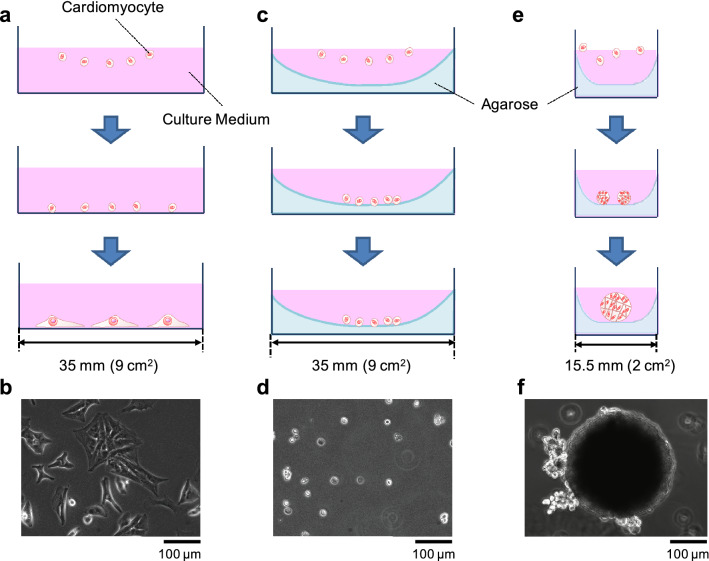
Formation of mouse primary cardiomyocyte clusters in agarose-coated wells. (**a**) Schematic drawing of the conventional dish cultivation of cardiomyocytes. The dispersed cells were cultured on the bottom of a 35-mm non-agarose-coated dish. After spread of the 2.0 mL of $5.0\times 10^{4}\, {\rm cells/mL}$ isolated single cardiomyocytes, the cells attached on the bottom of the 35-mm cultivation dish dispersedly. The cells started to beat 2–3 days after cultivation started. (**b**) A micrograph of dispersed cardiomyocytes in a 35-mm non-agarose-coated dish. (**c**) Schematic drawing of the cultivation of dispersed cells in a 35-mm agarose-coated dish. After spread of the 2.0 mL of $5.0\times 10^{4}\, {\rm cells/mL}$ isolated single cardiomyocytes, the cells dispersed on the bottom of the agarose layer in the agarose-coated 35-mm cultivation dish. Even after 2–3 days of cultivation, the cells remained isolated with a round shape, and no clusters formed on the bottom. (**d**) A micrograph of cardiomyocytes in an agarose-coated 35-mm cultivation dish. (**e**) Schematic drawing of the cultivation of dispersed cells in a 15.5-mm agarose-coated cultivation well (in a 24-well cultivation plate). After spread of the 1.0 mL of $5\times 10^{4}\hbox { cells/mL}$ isolated single cardiomyocytes, dispersed cells gathered and formed small clusters; finally, they gathered into a single large cluster in the 15.5-mm agarose-coated cultivation well. (**f**) A micrograph of a cardiomyocyte cluster in a 15.5-mm agarose-coated cultivation well.

First, we examined the procedure of cardiomyocyte cluster formation, employing the concave agarose bottoms of cultivation wells (agarose-coated wells) containing mouse primary cardiomyocytes. Without agarose coating, the cells attached dispersedly to the bottom of the 35-mm collagen-coated dish and spontaneously started to beat (upper schematic drawing in Fig. 1a and lower micrograph in Fig. 1b). In contrast, the cardiomyocytes cultivated in the agarose-coated wells did not attach to the bottom (upper schematic drawing in Fig. 1c and lower micrographs in Fig. 1d). However, the cells remained isolated dispersedly, and no clusters were formed on the flat agarose substrates in the wells. Hence, the narrower agarose-coated wells having a concave curvature of agarose at the bottom were prepared for cluster formation (schematic drawings in Fig. 1e). First, ${2.0}\,\upmu {\rm L/mm}^2$ of sol state 2.5% (w/v) agarose solution was poured into each well (inner diameter 15.5 mm) of the 24-well plates. After agarose gelled with a concave shape at the bottom of each well, 1.0 mL of $5.0 \times 10^{4}\, {\rm cells/mL}$ primary cardiomyocytes were seeded and incubated with mild shaking of the cultivation buffer in the well. After 2 days of cultivation, the cells were rolled on the bottom of the concave curvature of the agarose layer and formed a large single cardiomyocyte cluster at the small flat area at the center of each well of the 24-well plate (Fig. 1f).

**Figure 2:**
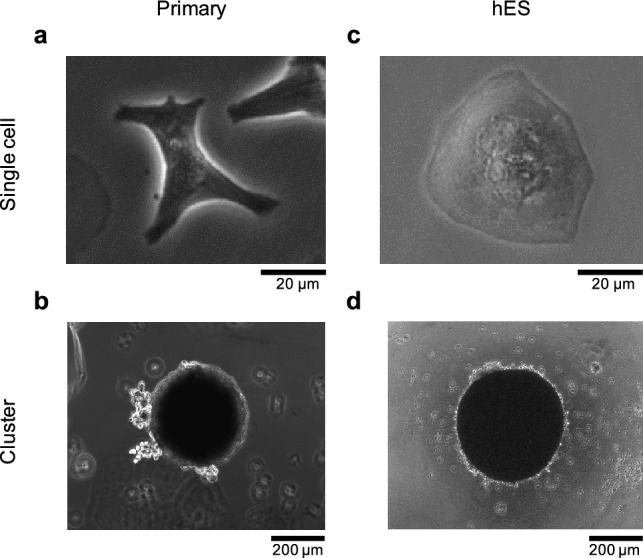
Micrographs of single cells and clusters of mouse primary and hES-derived cardiomyocytes. (**a**) Mouse primary cardiomyocytes (primary) in a 35-mm non-agarose-coated dish (single cell), (**b**) primary cells in a 24-well agarose-coated plate (cluster), (**c**) hES cardiomyocytes in a 35-mm non-agarose-coated dish (single cell), and (**d**) hES in a 24-well agarose-coated plate (cluster).

Next, we examined the cluster formation ability of human-stem-cell-derived cardiomyocytes (hES) in the same 24-well agarose-coated plates using the same method as described above. As shown in Fig. 2, after 2 days of cultivation, primary cells attached dispersedly in the collagen-coated 35-mm dish (Fig. 2a) and formed a large round cluster in the 24-well agarose-coated plate (Fig. 2b). Under the same conditions, hES cells were also attached dispersedly in the collagen-coated 35-mm dish (Fig. 2c), and a similar large round cluster of hES cardiomyocytes formed in the 24-well agarose-coated plate (Fig. 2d). The results indicate that the 24-well agarose-coated plate can work well for the formation of stable large cardiomyocyte clusters.

### Interbeat intervals of single cells and clusters for primary and hES cardiomyocytes

To investigate the relationship between the interbeat intervals (IBIs) of single cardiomyocytes and those of their clusters, we prepared single dispersed cells in collagen-coated 35-mm dishes and their clusters in 24-well agarose-coated plates. For single-cell cultivation, 2.0 mL of $5.0\times 10^{4}\hbox { cells/mL}$ mouse primary and hES cardiomyocytes were cultivated dispersedly in the collagen-coated dishes. Moreover, 1.0 mL of the same mouse primary and hES cardiomyocytes at $5.0\times 10^{4}\hbox { cells/mL}$ were used for cluster formation in the concave agarose-coated wells. Then, we measured six clusters and 73 single isolated cells for mouse primary cardiomyocytes, and 27 clusters and 125 single isolated cells for hES cardiomyocytes.

**Figure 3:**
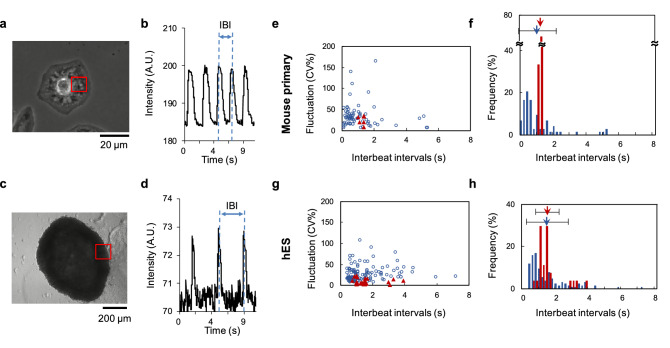
Analysis of interbeat interval (IBI) distribution of single and clustered mouse primary and hES cardiomyocytes. (**a**)–(**d**): Method of measuring interbeat interval (IBI) of single cardiomyocytes and clusters. Temporal change of luminance in the red square area for single cell (**a**) and cluster (**c**) caused by their beating was recorded, as shown in the time-course intensity profiles (**b**) and (**d**), respectively. IBIs of their beating were acquired from the time intervals between two neighboring peaks in the time-course intensity profiles. (**e**), (**f**): Distribution of IBIs of mouse primary cardiomyocytes. (**e**) The relationship between mean IBIs and fluctuations of beating [coefficient of variability (CV) of IBIs] of single isolated primary cardiomyocytes (blue open circles, n = 73) and primary clusters (red filled triangles, n = 6). (**f**) A histogram of all plots in (**e**). The blue filled bars indicate the frequency of IBIs of single cardiomyocytes; the blue arrow and the error bar indicate the corresponding mean value and standard deviation (SD) of single-cardiomyocyte IBIs, respectively. The red filled bars indicate the frequency of IBIs of clusters; the red arrow and the error bar indicate the corresponding mean value and SD of clusters. (**g**), (**h**): Distribution of IBIs in hES cardiomyocytes. (**g**) The relationship between mean IBIs and CV of IBIs in single isolated hES cardiomyocytes (blue open circles, n = 125) and hES clusters (red filled triangles, n = 27). (**h**) A histogram of all plots in (**g**). The blue filled bars indicate the frequency of IBIs of single cardiomyocytes; the blue arrow and the error bar indicate the corresponding mean values and SD of single cardiomyocytes, respectively. The red filled bars indicate the frequency of IBIs of clusters; the red arrow and the error bar indicate the corresponding mean value and SD of clusters.

As shown in Fig. 3a–d, the IBIs of clusters and isolated single cells were obtained from the displacements in their video images caused by their beating. To evaluate the relative fluctuation of IBIs, we utilized the coefficient of variability (CV) as described below:

1 $$\begin{aligned} \displaystyle CV (\%) = \frac{\sigma }{\mu } \times 100 \end{aligned}$$

where represents the ratio of the standard deviation $\sigma$(s) to the mean value $\mu$(s) of IBIs.

Figure 3e shows the distribution of the mean IBIs and their coefficient of variability (CV) for mouse primary single cells (blue open circles) and clusters (red filled triangles). Figure 3f also shows a histogram of IBIs of mouse primary single cells (blue) and clusters (red). Blue and red arrows indicate the mean IBIs of all single cells and clusters, and error bars represent their standard deviations (SDs), $1.11 \pm 1.17$ s and $1.25 \pm 0.168$ s, respectively. As shown in these graphs, the mean IBI distribution of single isolated cells was widely dispersed from 0.12 to 5.3 s, and 63% of dispersedly cultivated isolated single cells had shorter IBIs than the fastest-beating cluster.

For hES cells, Fig. 3g shows the distribution of the mean IBIs and the CV of IBIs in single cells (blue open circles) and clusters (red filled triangles), while Fig. 3h shows a histogram of the IBIs of single cells (blue) and clusters (red). Blue and red arrows indicate the mean IBIs of whole single cells and clusters, and their error bars represent SDs, 1.57 ± 1.18 s and $1.57 \pm 0.795$ s, respectively. The IBIs of hES single cells ranged from 0.44 to 7.2 s, while for clusters the range was 0.75 to 3.9 s; 12% of single isolated cells also had shorter IBIs than the fastest-beating hES cluster.

These results indicated the possibility that the shortest IBIs of clusters were longer than the IBIs of the constituent cells of the clusters. However, in this experiment, we did not compare the IBIs of constituent cells with those of the clusters directly. Hence, it remains a possibility that the clusters were formed from the slower-beating constituent cells, and the clusters’ IBIs were those of the fastest-beating constituent cells, even though 63% of isolated primary cells had shorter IBIs than the fastest-beating primary cluster.

We should also mention that the clusters’ fluctuation of IBI distribution was not smaller than the minimum fluctuation of single isolated cells. In our previous studies^16,17^, the CV of IBIs of cardiomyocytes decreased with increasing cell number. However, especially in primary clusters, the fluctuation of beating intervals was not reduced and was sometimes larger than that of the isolated single cardiomyocytes.

The recorded number of primary clusters was only six in this experiment. This does not actually mean that the number of clusters in the experiment on mouse primary cells was six, but most clusters ceased to beat during cultivation and only six of them kept beating. In contrast, hES clusters continued to beat for a long period and could be recorded.

### Interbeat intervals of isolated single cells acquired from hES clusters

The above results indicate that clusters were formed from the faster-beating constituent cells, but the clusters’ rhythm of beating did not follow that of the fastest-beating constituent cells. However, one concern remained. The results for the single isolated cells did not match the results of the constituent cells of the clusters, even though the same samples were used for the isolated single-cell cultivation and the cluster formation. Hence, to confirm that the IBIs of constituent cells were shorter than those of clusters, we measured the IBIs of the isolated constituent cells after recording the IBIs of their clusters.

**Figure 4:**
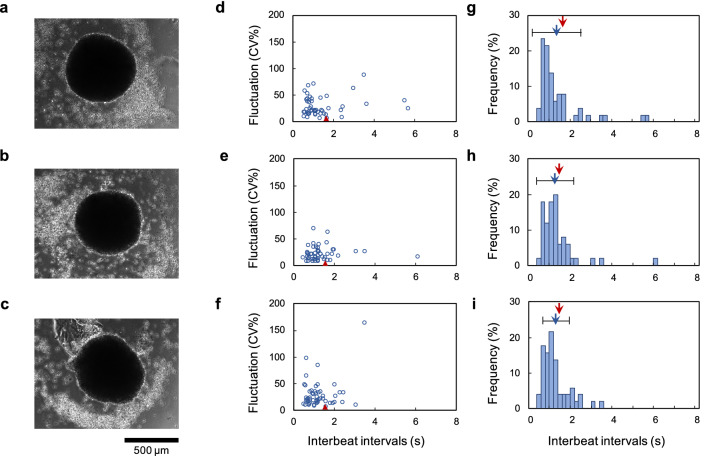
Distribution of IBIs and fluctuation of IBI distribution of the hES cardiomyocyte clusters and their constituent cells. (**a**)–(**c**): Micrographs of hES cardiomyocyte clusters. (**d**)–(**f**): Distribution of IBIs and the CV of IBIs in the clusters (**a**)–(**c**) and isolated constituent cells from each cluster (n=50 from among re-cultivated $1.0\times 10^{3}\hbox { cells}$). These plots (**d**)–(**f**) correspond to each cluster (**a**)–(**c**). The red filled triangles indicate the cardiomyocyte clusters, and the blue open circles indicate constituent cardiomyocytes of each cluster. Each cluster was measured 2 days after the beating started. Single cardiomyocytes were isolated from each cluster by trypsinization. IBIs of single constituent cardiomyocytes were measured 3 days after their isolation. Median and 95% confidence interval of single cardiomyocytes were 0.971 s and 0.825–1.25 s (**d**), 1.19 s and 1.00–1.28 s (**e**), and 1.12 s and 0.844–1.22 s (**f**), respectively. (**g**)–(**i**): Histograms of IBIs of each cluster and its isolated constituent cells. The blue filled bars indicate the ratio of frequency for single constituent cardiomyocytes; the blue arrows and error bars indicate the mean IBIs and SDs, and the red arrows also indicate the mean IBIs of clusters.

First, three hES cardiomyocyte clusters, formed from 1.0 mL of $5.0\times 10^{4}\hbox { cells/mL}$ in a 24-well agarose-coated plate (Fig. 4a–c), were cultivated for 3 days and their IBIs were recorded. Then, these clusters were trypsinized for isolation. Next, $1.0\times 10^{3}$ isolated single cells acquired from each cluster were re-cultivated dispersedly on a 35-mm collagen coated-tissue culture dish for 3 days and the IBIs of 50 isolated single cells of each cluster were recorded.

Figure 4d–f shows the IBIs and the CV of IBIs in isolated constituent single cells (blue open circles) and their clusters (red filled triangles). Figure 4g–i also shows histograms of these three samples. The blue filled bars indicate the frequency of IBIs in 50 re-cultivated single cardiomyocytes; the blue arrows and their error bars indicate the corresponding mean value of 50 cells and their SDs, and were $1.42 \pm 1.11$ s (Fig. 4g), $1.38 \pm 0.938$ s (Fig. 4h), and $1.28 \pm 0.652$ s (Fig. 4i). The red arrows indicate the mean IBIs of each cluster, which were 1.62 s (Fig. 4g), 1.53 s (Fig. 4h), and 1.54 s (Fig. 4i).

These plots also show that the majority of isolated single constituent cardiomyocytes had shorter IBIs than their clusters. Statistical analysis indicated that the 95% confidence interval of the median IBI of the single constituent cardiomyocytes was 0.825–1.25 s in Fig. 4d. This means that at least 50% of the constituent cells had an IBI shorter than 1.25 s. As the mean IBI of the cluster was 1.62 s, more than 50% of the constituent cardiomyocytes were expected to beat faster than the cluster. The IBI distributions of single cardiomyocytes in Fig. 4e,f also indicate that the 95% confidence intervals of the median IBI of the constituent single cardiomyocytes were 1.00–1.28 s and 0.844–1.22 s, respectively. As the mean IBIs of their clusters were 1.53 s and 1.54 s, more than 50% of constituent cells in these two clusters also had IBIs that were shorter than these clusters’ IBIs.

We also examined the influence of trypsinization on the IBIs of individual cardiomyocytes. We compared the IBIs of the dispersed single cardiomyocytes between before and after trypsinization. First, we measured the IBIs of single cardiomyocytes (n = 50 among $1.0\times 10^{3}\hbox { cells}$) 3 days after cultivation in a 35-mm dish; then, we added trypsin-EDTA to the dish and incubated it for 8 min at ${37}^{\circ }\hbox {C}$. Next, we rinsed the treated cells, cultivated them again for 3 days in another 35-mm dish, and measured their IBIs (n = 50).

**Figure 5:**
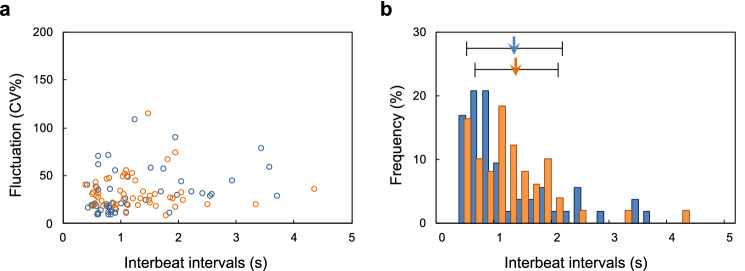
Influence of trypsinization on interbeat intervals in dispersed individual hES cardiomyocytes. (**a**) Distribution of the IBIs and the CV of IBIs in single hES cardiomyocytes before and after trypsinization. The blue open circles indicate the single hES cardiomyocytes (n = 50) before trypsinization. The orange open circles indicate the single cardiomyocytes (n = 50) after trypsinization. (**b**) Histograms of IBIs of hES single cardiomyocytes before and after trypsinization. The blue filled bars indicate the mean IBIs of single cardiomyocytes before trypsinization; the blue arrow and error bar indicate their mean value and SD. The orange filled bars indicate the frequency of mean IBIs of trypsinized single cardiomyocytes; the orange arrow and error bar indicate their mean value and SD.

Figure 5a shows the distributions of the IBIs and the CV of IBIs in single hES cardiomyocytes before and after trypsinization. The blue open circles indicate the single cardiomyocytes before trypsinization, while the orange open circles indicate them after trypsinization. The distributions of the plots of the two samples showed similar tendencies, with no noticeable difference between them ($p=0.31$, 95% confidence interval: 0.36 to 0.091), which indicates that the trypsinization did not influence the mean IBIs of single hES cardiomyocytes.

For statistical confirmation of this, we re-plotted these results. Figure 5b shows histograms of the IBI distributions of single cardiomyocytes before and after trypsinization. The blue filled bars indicate the frequency of IBIs of single cardiomyocytes before trypsinization; the blue arrow and error bar indicate the corresponding mean value and SD of those cells ($1.26 \pm 0.848$ s). The orange filled bars indicate the frequency of IBIs of trypsinized single cardiomyocytes, and the orange arrow and error bar indicate the corresponding mean value and its SD ($1.30 \pm 0.741$ s).

We also statistically compared these isolated constituent cells with the single hES cardiomyocytes (plotted in Fig. 3g) using the Mann–Whitney U test. The results showed that the *p* value and 95% confidence interval of each cluster sample were $p=0.56$, 0.12 to 0.24 (Figs. 3g and 4d), $p=0.88$, 0.19 to 0.20 (Figs. 3g and 4e), and $p=0.69$, 0.14 to 0.27 (Figs. 3g and 4f), respectively. These results indicate that there was no significant difference of IBI distribution between the isolated single hES cells cultivated dispersedly and the single constituent cells acquired from clusters subjected to trypsinization.

### Interbeat intervals of two clusters after their connection and subsequent separation

The above results show that the IBIs of clusters were within the IBIs of spontaneously beating constituent single cells and were also longer than that of the fastest-beating constituent cardiomyocyte. If isolated cardiomyocytes are connected, under the rule of “overdrive suppression,” the IBIs of the clusters should follow that of the fastest-beating constituent cardiomyocyte. However, the IBIs of the clusters were near the mean value of the single cardiomyocytes. These single cells-to-cluster results suggest the possibility that the cluster-to-cluster connection also does not match what would be expected under “overdrive suppression,” but that a synchronized IBI that is the mean of those of two clusters develops after their contact.

**Figure 6:**
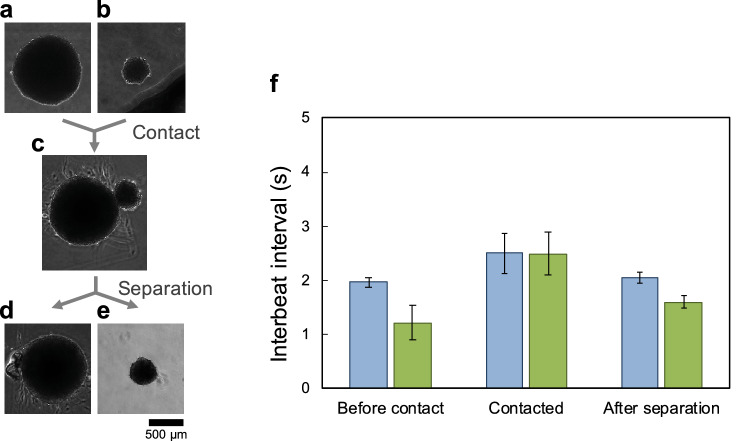
Distributions of interbeat intervals (IBIs) and fluctuations of the two hES cardiomyocyte clusters before and after their connection and after re-separation. (**a**)–(**e**): Micrographs of cardiomyocyte clusters. Micrographs of the large cluster (**a**) and small cluster (**b**) before contact. These clusters were measured when they had been cultivated for 7 days. The two hES cardiomyocyte clusters were connected (**c**). The measurement was performed 3 days after the two clusters contacted each other. Micrographs of the large cluster (**d**) and small cluster (**e**) after separation. The measurements were taken within 5 min of separation. (**f**): Distribution of IBIs and fluctuations of two clusters before contact, during contact, and after separation. Blue filled bar and error bar indicate the mean IBIs and SD of the large cluster. Green filled bar and error bar indicate the mean IBIs and SD of the small cluster.

To confirm this possibility, we connected two spontaneously beating clusters and compared their beating synchronization behavior between before and after their connection. Two hES cardiomyocyte clusters were formed from 1.0 mL of $1.0\times 10^{5}\hbox { cells/mL}$ for the large cluster, and 1.0 mL of $1.0\times 10^{4}\hbox { cells/mL}$ for the small cluster, on the agarose concave structure in a 24-well plate. Figure 6a,b shows micrographs of the large and small clusters before their connection. After 7 days of cultivation, we recorded their IBIs as the “before contact” data. Then, using a micropipette, we manipulated the small cluster to move it adjacent to the large cluster to induce contact between these two clusters on the culture dish (Fig. 6c). After 3 days of cultivation after their contact, we recorded the synchronized IBIs of these two contacting clusters. Finally, we separated these two clusters using ophthalmic scissors and recorded the IBIs of these two clusters as the “after separation” data (Fig. 6d,e).

Figure 6f shows the distribution of IBIs and the CV of IBIs in two clusters under these three conditions: before contact, during contact, and after separation. The blue bars and green bars show the mean IBIs of the large and small clusters, respectively. The uncertainties are given by the SDs (error bars in Fig. 6f). Before contact of the two clusters, the mean IBIs and SDs of the large cluster and small cluster were 1.97 ± 0.0926 s and $1.21 \pm 0.312$ s, respectively (Fig. 6f (before contact)). After connection of the two clusters, they were synchronized to the same mean IBI and SD, 2.49 ± 0.379 s, which was 1.5 times longer than the IBI of the large cluster and was also more than twice as long as the IBI of the small cluster [Fig. 6f (during contact)]. After separation of the two clusters, the mean IBIs and SDs of the large cluster and the small cluster were recovered to shorter IBIs with smaller SDs, $2.05 \pm 0.0955$ s and $1.59 \pm 0.110$ s, respectively (Fig. 6f (after separation)).

The results showed that the synchronized IBIs of the two connected clusters also contradicted the faster firing regulation or “overdrive suppression.” Moreover, they were not intermediate between the IBIs of the large cluster and the small cluster. This means that the emergence of slower synchronized beating from faster-beating elements was observed. This phenomenon cannot be explained by “overdrive suppression” or by the conventional oscillation models, even though the fluctuation–dissipation theorem is considered in the oscillation synchronization phenomenon.

Two characteristic phenomena were observed in this synchronization of two clusters. One is that the fluctuation of the synchronized IBI distribution of the connected two clusters increased compared with that of the isolated clusters. This contrasts with the conventional understanding of the phenomenon of cardiomyocyte network synchronization. In our previous reports^16,17,20^, an increase in the number of connected cardiomyocytes contributed to decrease the fluctuation of IBI and increase its stability. However, upon cluster-to-cluster connection, this contribution of the number of connected cardiomyocytes to increased stabilization was not observed, which indicates that the “number increases stabilization” rule does not always dominate the synchronization of spontaneously beating cardiomyocytes. The other characteristic phenomenon is that the two synchronized clusters recovered to shorter IBIs in both of the two isolated clusters after their separation. The CV of IBIs of two isolated clusters was also decreased compared with that under the conditions of synchronized beating. These characteristics might explain that the synchronized slower beating of two clusters was not formed by harmonic oscillation synchronization but by the competitive suppression between two clusters.

### Can overdrive suppression explain the synchronized beating intervals of cardiomyocyte clusters?

Using the simple concave agarose structure-based clustering method, we examined whether faster firing regulation can explain the synchronized beating of cardiomyocyte networks. We formed cardiomyocyte clusters from isolated single cardiomyocytes and compared the IBIs of single isolated cells and these clusters. As shown in Figs. 3 and 4, the single cardiomyocytes showed a wider distribution of IBIs. However, once those cells were gathered into a cluster, the cluster had the original stable IBIs with a lower CV of IBIs than the isolated single cells. It had longer IBIs than the dispersed isolated single cardiomyocytes having shorter IBIs (Fig. 3). To confirm this, we also separated the constituent cells from the clusters and confirmed that the single constituent cells beat faster than their clusters did (Fig. 4). Hence, the faster-beating cells do dominate the IBIs of the clusters, which is discordant with the faster firing regulation of heart beating^1^ also known as “overdrive suppression”^4^.

The firing of spontaneously beating cardiomyocytes is induced electrically by the threshold potential of the cell membrane being reached with a very small net inward current of ions across it, which gives rise to action potential. As the membrane potential travels from the fastest firing cells to the resting (slower intrinsic beating) cells before their spontaneous firing, the faster firing regulation is thought to dominate the beating intervals of cardiomyocyte networks. Hence, from this local membrane potential-based perspective, our experimental results do not match the undisputed, specific membrane potential-based faster-firing regulation process.

In our previous on-chip studies, we found that the synchronization of cardiomyocytes was dominated by regulation of the lower CV of IBI, rather than the faster firing regulation. This was determined experimentally using on-chip single-cell-based constructive synchronization observation^16,17,19^, and theoretically with in silico model simulations employing the “fluctuation–dissipation theorem” in the phase-couple synchronization model^20^. In this study, the single cells-to-cluster synchronization also showed the same tendency as our previous studies; IBIs of clusters were not regulated by the faster-firing cells within the cluster. The lower fluctuation regulation appears to be suitable to explain the synchronization behavior of clusters. Because the lower fluctuation regulation reflects the hysteresis of the manner of connection of cells during clustering, it is caused by the community effect of cardiomyocytes; that is, the stability of cardiomyocyte beating increased with the increasing number of cells within the network.

In our previous report^20^, we discussed the limitation of conventional phase equations like the Kuramoto model to explain the regulation of the lower CV of IBIs of cardiomyocyte networks and included the “fluctuation–dissipation theorem” in the phase-couple synchronization model to satisfy the lower CV of IBI regulation experimental results. If the cardiomyocytes are well described only by phase equations of the Kuramoto model, a cluster’s IBI equals the mean value of the IBIs of its constituents because:

2 $$\begin{aligned} \sum _{i=1}^N\frac{1}{N}\frac{d\phi _i}{dt}=\frac{1}{N}\sum _{i=1}^N \left\{ \omega _i+\frac{K}{N}\sum _{j=1}^N sin(\phi _j-\phi _i)\right\} =\frac{1}{N}\sum _{i=1}^N \omega _i =<\omega >\end{aligned}$$

where $\phi _i$ is the phase variable indicating the periodically changing state of the *i*-th cell and $\omega _i$ is the natural angular velocity of the *i*-th cell. *N* is the number of constituent cells of a cluster, and *K* is a constant indicating the strength of connections between constituent cells. We assume that *K* is sufficiently strong so that all phases, $\phi _i$, are locked into a certain type of distribution. Mutual interaction terms are dropped because *sin* is an odd function. Upon referring to Fig. 4, it is suggested that the gathering and beating synchronization of cardiomyocytes derived from primary and hES cardiomyocytes can be well described by simple phase equations because the mean of the single cells’ IBIs and the clusters’ IBIs are almost the same.

Moreover, as shown in Fig. 3f, IBIs of the primary cardiomyocyte clusters converged to a single sharp peak with a smaller SD. This means that the manner of cluster formation from a variety of different beating elements does not influence the IBIs of clusters. In other words, when the clusters are not sensitive to the process or order of cell-to-cell connection from single cells with different patterns of beating to form clusters, the IBIs of clusters should converge on the mean IBI of all of their constituent cells, as described in the above equation [Eq. (2)]. However, if the initial value and connection process does not make any difference to the characteristics of clusters, the results of cluster-to-cluster connection should also follow the mean value of these two clusters, the same as the above interpretation. Hence, the results of cluster-to-cluster formation indicate that the clustered cardiomyocytes should be different species from the isolated single cells. In addition, these sufficiently large clusters do not satisfy the rule that “cluster formation from a variety of different beating elements does not influence the IBIs of clusters” or “overdrive suppression.” In particular, the recovery process of shorter IBIs of two clusters after their separation supports the idea that some memorable individuality is conserved in the clusters when they reach a sufficient size.

The findings reported in this paper indicate the importance of the community effect of cells, which cannot be explained simply by expanding the knowledge from single-cell studies on membrane potentials, as described in our previous patch-clamp membrane potential measurement of clustered, spontaneously beating cardiomyocytes^24^. Clarification of the rules determining the obtained findings based on the higher complexity of cell networks should lead to the development of well-designed quasi-in vivo models as the community effect of cardiomyocytes.

In this study, we measured only homogeneous cell clusters, so the results and interpretation presented here should be considered as-yet unproven. In other words, as this study is just limited to IBI analysis of a network composed of cells with potentially different phenotypes at different stages of maturation, more accurate quality and quantity control of cells is desirable for further experiments. To overcome these limitations of homogeneity of prepared cells in practical experiments, in silico simulation should be one potential solution. The influence of contamination with different types of cell such as fibroblasts on the results should also be considered as a next step because such cells have been examined using on-chip cell-network assays, with the results indicating that the fibroblasts suppressed the synchronization ability^30,31^. Further study using a variety of other sources of cardiomyocytes and other cells like fibrobkasts with on-chip electrophysiological measurements would deepen our understanding of the slower synchronization phenomenon and its mechanism.

## Conclusion

In conclusion, we examined the dominant rule determining the synchronized beating intervals of cardiomyocyte clusters of mouse primary and hES cardiomyocytes by comparing the IBI and fluctuation characteristics of these single isolated cells and those in clusters. We also examined cluster-to-cluster connections for their synchronization. The results showed that the clusters directly formed from single cells synchronized not to the fastest-beating constituents but instead reached the mean IBI regardless of the order in which the different cells congregated into the cluster. Moreover, cluster-to-cluster formation emerged the slower synchronization beating behavior than original clusters. We confirmed that the beating synchronization of clusters does not match the faster firing regulation of heart beating or “overdrive suppression,” but can be explained by some other emergent synchronization regulatory pattern as a community effect of cells.

## Methods

This study was carried out in strict accordance with the Act on Welfare and Management of Animals of the Ministry of the Environment, Japan. All animal experiments and protocols were approved by the Animal Experiment Committee of Waseda University (permission numbers: 2019-A072 and 2020-A022) and adhered to guidelines (ARRIVE 2.0) and regulations for animal experimentation.

### Cells

Two types of cardiomyocyte were used for this experiment. Embryonic mouse primary cardiomyocytes (primary) were isolated and purified from 14-day-old ICR mouse embryos using a modified version of a method described in our previous reports^16,17^. In brief, 14-day-old ICR mice were purchased from Tokyo Laboratory Animals Science Co., Ltd. (Tokyo, Japan). The embryos were rapidly removed from pregnant mice anesthetized with pentobarbital sodium salt (15 mg/kg; Nacalai Tesque, Inc., Kyoto, Japan), which was intraperitoneally administered to the pregnant mice, and isoflurane (${<}$ 2%; Wako Pure Chemical Industries, Osaka, Japan), which was volatilized by a vaporizer (NARCOBIT-E(II); Natsume Seisakusho Co., Ltd., Tokyo, Japan). The mouse embryos were removed from the uterus under anesthesia. The hearts of the embryos were obtained by trimming the embryos with tweezers and scissors and washed with phosphate-buffered saline (PBS; Takara Bio Inc., Kusatsu, Shiga, Japan) containing 0.9 mM $\hbox {CaCl}_2$ and 0.5 mM $\hbox {MgCl}_{2}$ to induce heart contraction and remove corpuscle cells. The hearts were then transferred to PBS without $\hbox {CaCl}_{2}$ and $\hbox {MgCl}_{2}$ and the ventricles were separated from the atria and minced into 1-$\hbox {mm}^3$ pieces with scissors. After that, they were incubated at ${37}^{\circ }\hbox {C}$ for 30 min in PBS containing 0.2% collagenase (Wako) to digest the ventricular tissue. After this digestion step had been repeated twice, the cell suspension was transferred to primary cultivation buffer (Dulbecco’s modified Eagle’s medium: DMEM; Invitrogen, Carlsbad, CA, USA) supplemented with 10% heat-inactivated fetal bovine serum (FBS; Invitrogen), 100 U/mL penicillin, and ${100}\,\upmu \hbox {g/mL}$ streptomycin (Invitrogen) at ${4}^{\circ }\hbox {C}$. In the subsequent experiments, the above medium was used to handle embryonic mouse primary cardiomyocytes. The cells were filtered through a ${40}\,\upmu \hbox {m}$ nylon mesh cell strainer (BD Bioscience, Franklin Lakes, NJ, USA) to remove debris that could not be digested and then centrifuged at 180 g for 5 min at room temperature. After precipitation of the cells, they were resuspended gently in Cell Banker I (Takara Bio Inc.), frozen, and stored in liquid nitrogen. For the measurement experiments, the stored frozen cells were thawed and cultivated in a primary cultivation buffer.

Human embryonic stem cell-derived cardiomyocytes (hES) (hES-CMCTM002, hES cell line SA002) were purchased from Cellectis (Gothenburg, Sweden)^32,33^. The hES cardiomyocytes were cultivated in hES-cultivation buffer (DMEM, high glucose; GlutaMAX) and pyruvate (Invitrogen) supplemented with 20% heat-inactivated fetal FBS, 100 U/mL penicillin, ${100}\,{\upmu \hbox {g/mL}}$ streptomycin, 1.0% nonessential amino acids (Invitrogen), and 0.1 mM $\beta$-mercaptoethanol (Invitrogen).

### Cell culture

For the pre-cultivation, a collagen-coated 35-mm dish was prepared with Cellmatrix Type 1-C (Nitta Gelatin Inc., Osaka, Japan) diluted 10-fold with 1 mM HCl. ${200}\,{\upmu \hbox {L}}$ of the collagen solution was spread on a tissue culture dish (AGC Techno Glass Co., Ltd., Shizuoka, Japan) evenly and dried up. Then, it was thoroughly washed with cultivation buffer. Two types of cryopreserved cell (primary and hES cardiomyocytes) were thawed and thoroughly washed with cultivation buffer. The number of surviving cells was determined with an automated cell counter (TC-20; Bio-Rad, Hercules, CA, USA). To observe the behavior of single cells, 2 mL of $2.0\times 10^{5}\hbox { cells/mL}$ of cardiomyocyte resuspensions (primary and hES cardiomyocytes) were cultivated in a collagen-coated 35-mm dish for 3 days at ${37}^{\circ }\hbox {C}$ in 5% $\hbox {CO}_2$. Then, the cardiomyocytes were isolated from the collagen-coated dishes with 0.25% trypsin-EDTA (Invitrogen) and harvested. After 4 min of incubation at ${37}^{\circ }\hbox {C}$, the digestion reaction was stopped by each cultivation buffer containing serum and thoroughly washed. The cardiomyocytes were resuspended in each cultivation buffer at a concentration of $5.0\times 10^{4}\hbox { cells/mL}$.

For the dispersed single-cell cardiomyocyte experiments, 2.0 ml of $5.0\times 10^{4}\hbox { cells/mL}$ cardiomyocyte resuspensions (primary and hES cardiomyocytes) were cultivated in a collagen-coated 35-mm dish for 3 days at ${37}^{\circ }\hbox {C}$ in 5% $\hbox {CO}_2$ and their spontaneous beating was observed.

For the cluster experiments, cardiomyocyte clusters were formed in a concave agarose structure and their spontaneous beating was observed. First, ${500}\,{\upmu \hbox {L}}$ of a melted 2.5% agarose (Promega, Madison, WI, USA) solution was applied to the bottom of 24-well microplates (AGC Techno Glass Co., Ltd.) and incubated at room temperature for 2 h for gelation. Next, the concave-shaped agarose structures, formed on the bottoms of the microplates, were washed and equilibrated for 30 min with each cultivation buffer. Then, 1.0 ml of $5.0\times 10^{4}\hbox { cells/mL}$ of cardiomyocyte suspension was applied to each of 24 wells, cultivated for 3 days at ${37}^{\circ }\hbox {C}$ in 5% $\hbox {CO}_2$, and the spontaneous beating of the formed clusters was observed.

### Isolation and re-cultivation of single cardiomyocytes from clusters

After measurement of the spontaneous beating, cardiomyocyte clusters were digested with 0.25% trypsin-EDTA (twice for 4 min at ${37}^{\circ }\hbox {C}$) to acquire the constituent cardiomyocytes. The acquired isolated single cardiomyocytes were dropped into a collagen-coated 35-mm dish and cultivated at ${37}^{\circ }\hbox {C}$ in 5% $\hbox {CO}_2$ for 3 days to observe their spontaneous beating.

### Contact and separation of two cardiomyocyte clusters

Large and small clusters were formed as follows. Large clusters were formed from 1.0 mL of $1.0\times 10^{5}\hbox { cells/mL}$ cardiomyocytes on the agarose concave structures in a 24-well plate, while small clusters were formed from 1.0 mL of $1.0\times 10^{4}\hbox { cells/mL}$ cardiomyocytes on the same agarose concave structures in a 24-well plate. A large cluster was placed on the bottom of a collagen-coated 35-mm dish, and then a small cluster was manipulated with a micropipette (CellTram; Eppendorf, Hamburg, Germany) to make it contact the large cluster. After 3 days of cultivation and observation of beating, the two connected clusters were separated back into the original small and large clusters by cutting with ophthalmic scissors (Fine Science Tools Inc., North Vancouver, BC, Canada) and their beating was observed.

### Interbeat interval (IBI) recording

Interbeat intervals (IBIs) are the time intervals between individual beats of cardiomyocytes and were measured as the time course of luminance of cardiomyocyte images from beat to beat caused by their contraction with ImageJ (US National Institutes of Health, Bethesda, MD, USA). Incubated cardiomyocyte clusters or single cells were set under an inverted optical microscope (IX-71; Olympus, Tokyo, Japan) equipped with a cooled charge-coupled-device (CCD) camera recording system (ORCA-ER; Hamamatsu Photonics, Hamamatsu, Shizuoka, Japan). Their temporal displacement of a portion of the cell surface caused by the spontaneous contraction intervals was measured every 1/30 s with ImageJ. We regarded these intervals of time-course displacement as IBIs.

### Statistical analysis

All statistical values of IBIs and their fluctuations are presented as mean±standard deviation (SD) of 1-min recorded IBIs (unless stated otherwise). All IBIs of single cells and clusters were evaluated using the Mann–Whitney U test when comparing multiple groups. $p<0.05$ was considered statistically significant and the 95% confidence interval was also regarded as statistically independent and identically distributed. U test was performed using R (Ver. 4.0.3; R Core Team, R Foundation for Statistical Computing, Vienna, Austria).
